# Hydrophobicity and Charge Shape Cellular Metabolite Concentrations

**DOI:** 10.1371/journal.pcbi.1002166

**Published:** 2011-10-06

**Authors:** Arren Bar-Even, Elad Noor, Avi Flamholz, Joerg M. Buescher, Ron Milo

**Affiliations:** 1 Department of Plant Sciences, The Weizmann Institute of Science, Rehovot, Israel; 2 Institute of Molecular Systems Biology, ETH Zurich, Zurich, Switzerland; University of Wisconsin-Madison, United States of America

## Abstract

What governs the concentrations of metabolites within living cells? Beyond specific metabolic and enzymatic considerations, are there global trends that affect their values? We hypothesize that the physico-chemical properties of metabolites considerably affect their *in-vivo* concentrations. The recently achieved experimental capability to measure the concentrations of many metabolites simultaneously has made the testing of this hypothesis possible. Here, we analyze such recently available data sets of metabolite concentrations within *E. coli*, *S. cerevisiae*, *B. subtilis* and human. Overall, these data sets encompass more than twenty conditions, each containing dozens (28-108) of simultaneously measured metabolites. We test for correlations with various physico-chemical properties and find that the number of charged atoms, non-polar surface area, lipophilicity and solubility consistently correlate with concentration. In most data sets, a change in one of these properties elicits a ∼100 fold increase in metabolite concentrations. We find that the non-polar surface area and number of charged atoms account for almost half of the variation in concentrations in the most reliable and comprehensive data set. Analyzing specific groups of metabolites, such as amino-acids or phosphorylated nucleotides, reveals even a higher dependence of concentration on hydrophobicity. We suggest that these findings can be explained by evolutionary constraints imposed on metabolite concentrations and discuss possible selective pressures that can account for them. These include the reduction of solute leakage through the lipid membrane, avoidance of deleterious aggregates and reduction of non-specific hydrophobic binding. By highlighting the global constraints imposed on metabolic pathways, future research could shed light onto aspects of biochemical evolution and the chemical constraints that bound metabolic engineering efforts.

## Author Summary

What governs the identity and concentrations of metabolites within living cells? The first part of this question has received much attention. Organisms were found to qualitatively prefer hydrophilic and charged metabolites, a phenomenon that was explained to be a result of constraints imposed by contemporary as well as archaic metabolism. However, among the metabolites that are used, a quantitative preference has never been analyzed systematically. Here we use the most comprehensive data sets of metabolite concentrations available to explore such trends. We find that in various organisms and growth conditions, living cells minimize the concentrations of non-polar, un-charged metabolites. More specifically, metabolites' hydrophobicity alters concentrations by two orders of magnitudes on average and explains up to half of the variation of metabolite concentrations within cells. We suggest that this can be attributed to an evolutionary pressure to avoid an unspecific hydrophobic effect: the preference of hydrophobic surfaces in an aqueous environment to adhere to other hydrophobic surfaces. Our findings shed light on the evolution of the internal makeup of living cells and can assist in establishing metabolic models that support synthetic biology and metabolic engineering efforts.

## Introduction

Living cells exhibit a preference towards certain types of metabolites. Many of these tendencies can be explained as consequences of chemical constraints imposed on metabolism. For example, the cellular ubiquity of charged metabolites, like those containing phosphoryl or carboxyl groups, is attributed to increasing solubility and decreasing leakage through the membrane [1].

Several studies suggest that contemporary structural preferences can be attributed to characteristics of archaic metabolism [2], [3]. For example, it has been suggested that positively charged surfaces played a central role in archaic metabolism, selecting for negatively charged molecules, mainly carboxylates and phosphates [3], [4]. Such conditions also favored water-eliminating polymerization reactions, resulting in the formation of large biomolecules like those that make up most of the biomass in contemporary cells [3]. In addition, early energy demands probably involved the use of iron and sulfur [3], [4], elements that still play a central role in living organisms. Focusing on carbon fixation, the availability of various reduced metals and volatile C1 compounds in the highly reduced early environment probably account for the structure of some of the contemporary carbon fixation pathways [5].

In this study we explore whether the qualitative preferences for specific types of metabolites represent a systematic, quantitative trend across multiple organisms. We suggest that a quantitative perspective on the chemical preferences of living cells could help elucidate the evolutionary forces shaping the structure of metabolic systems, facilitate genome-scale metabolic reconstructions and advance the design and implementation of novel metabolic pathways [6].

A previous study [7] demonstrated that the specific chemical groups composing metabolites explain a fraction of the variance in their concentrations. However, this previous work collected concentration values from separate sources, each employing different conditions and measurements techniques. In our study we use data sets of simultaneously measured concentrations of dozens of metabolites. We report a comprehensive correlation analysis between physico-chemical parameters of metabolites and their *in-vivo* concentrations. We find consistent trends which suggest that, beyond specific metabolic effects on concentrations, such as the kinetics of the enzymes producing and consuming a metabolite, there are global evolutionary tendencies that shape the internal makeup of living cells.

## Results

We employed two large data sets of measured metabolite concentrations in *E. coli* which represent the most comprehensive data sources currently available (Bennett *et al*. [8], containing 93 metabolites and Ishii *et al*. [9], 108 metabolites). To strengthen our analysis we have further used five smaller data sets: three are from *S. cerevisiae* (Ewald *et al*. [10], 29 metabolites, Fendt *et al*. [11], 29 metabolites, and Kummel *et al*. [12], 33 metabolites); one from *B. Subtilis* (Kleijn *et al*. [13], 35 metabolites) and another contains measurements of the 20 common amino acids in human muscle (Bergstorm *et al*. [14]). Most of these data sets contain at least three different conditions in which concentrations were measured. Overall, 21 conditions were analyzed independently. The full concentration data is given in the Dataset S1.

**Figure 1:**
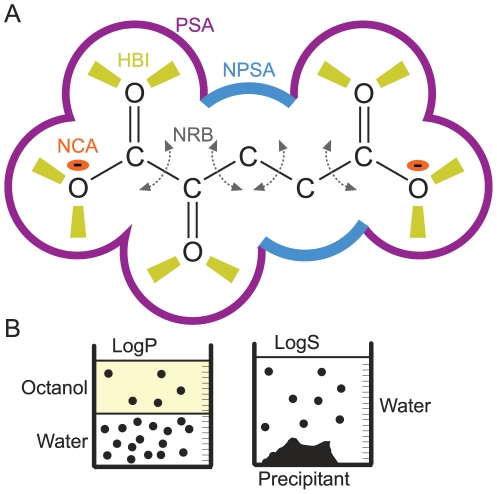
Schematic representation of physico-chemical parameters of metabolites (Materials and Methods), exemplified using 2-ketoglutarate. (a) Purple - polar surface area (PSA, oxygen and nitrogen atoms that are able to form hydrogen bonds, including hydrogen atoms attached to them). Blue - non-polar surface area (NPSA) which contributes to the hydrophobic effect. Yellow trapezes represent hydrogen bonds that the molecule can form with the solvent or with other solute molecules (HBI - hydrogen bond inventory). Charges are marked by red ellipses (NCA – number of charged atoms). Curved, dashed grey arrows correspond to rotatable bonds (NRB – number of rotatable bonds). (b) LogP (left) is the logarithm of the equilibrium ratio of concentrations of a metabolite in the two phases of a mixture of octanol and water. LogS (right) is the logarithm of the water solubility. See Materials and Methods for details on the calculation of these parameters.

We analyzed various physico-chemical parameters associated with the different metabolites, including molecular mass (MW), polar surface area (PSA), non-polar surface area (NPSA), number of charged atoms (NCA), hydrogen bond inventory (HBI), number of rotatable bonds (NRB), solubility in water (LogS) and lipophilicity (LogP, the ratio of the equilibrium concentrations of a compound in octanol and water), as shown in Figure 1 (Materials and Methods). We focus our discussion on small metabolites (MW≤300 Da) as we find that these show the most prominent correlations. This group contains most (≥80%) of the metabolites in each of the original data sets. The excluded metabolites includes mostly co-factors (e.g. NADPH, ATP etc), which are expected to be subject to a different and stronger set of selective pressures, alongside phosphorylated nucleotides and CoA substituted compounds. Notably, the qualitative trends we describe below also persist in the full data set, albeit less clearly (Figure S1).

**Figure 2:**
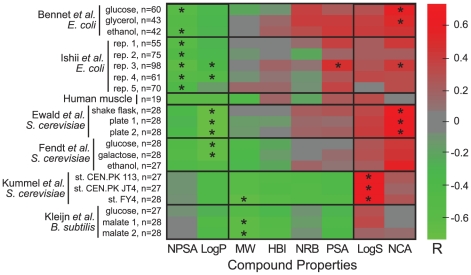
Correlation (R) between the logarithm of metabolites concentrations in each data set and the physico-chemical parameters of metabolites. Only metabolites with MW<300 were included in this analysis (see Text S1 and Figure S1). We computed the p-value of each R^2^ and determined its significance, as explained in the Methods. A correlation that was found to be significant (false discovery rate of 0.01, see Methods) is denoted by *. Parameters abbreviations are as in Figure 1.

**Figure 3:**
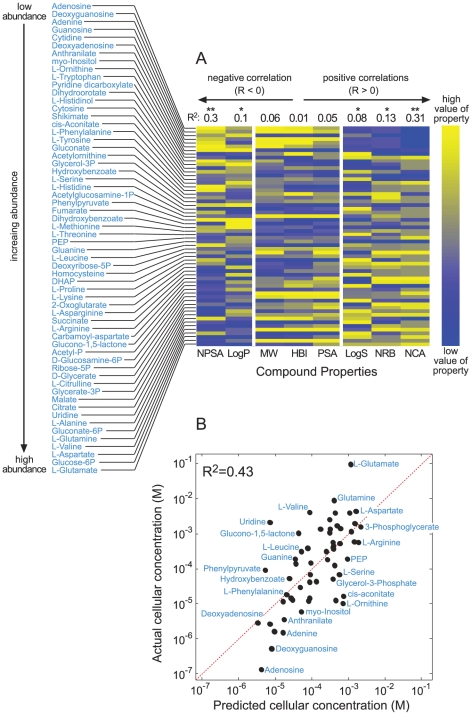
Physico-chemical parameters significantly correlate with the logarithm of the metabolite concentrations in glucose grown *E. coli*, as measured by Bennett *et al*. [**8**]. (a) Metabolites are ordered (top to bottom) by increasing concentration. Physico-chemical parameters are ordered based on their correlation with concentrations, from the most negative correlation on the left to the most positive correlation on the right. Compound properties were normalized by subtracting the mean and dividing by the standard deviation, enabling consistent color coding of their values. R^2^ values are given at the top of the columns. p-values were calculated as described in the Methods, where ** correspond to a p-value<10^-4^ and * to a p-value<10^-2^. Parameter abbreviations are as in Figure 1. (b) A linear regression using NPSA and NCA explains about half of the variability in metabolites concentration, as shown by a Log-Log correlation between the expected and measured concentrations.

In Figure 2 we show the level of correlation between the physico-chemical parameters analyzed and the logarithm of metabolite concentrations for each of the 21 experimental conditions. Even though the data sets are known to be noisy for experimental reasons we find that some parameters are consistently correlated with metabolite concentrations whereas others show no consistent correlation. The non-polar surface area (NPSA), LogP, LogS and the number of charged atoms (NCA) correlate with concentrations across the data sets and conditions (Figure 2) and point to a systematic phenomenon: the concentrations of non-polar, un-charged metabolites are significantly lower within cells. Specifically, in the two large data sets (Figures 3A and S2), metabolite concentrations decrease on average ∼100 fold with increasing NPSA. In the *S. cerevisiae* data sets, concentrations increase ∼100 fold with decreasing LogP or increasing LogS (depending on the data set, Figure 2). The lower correlation observed in the data set of Kleijn *et al.* can be attributed to the multiple analytical platforms that the authors used for the measurement of the metabolites, which might introduce different experimental biases.

In Bennett *et al*. [8], the most reliable and comprehensive data set (see below), we find that a regression analysis using only NPSA and NCA accounts for almost half of the variation in metabolite concentrations within the cell (R^2^ = 0.43, glucose-fed *E. Coli*, Figure 3B). Moreover, while ∼55% of metabolites' concentrations are within one order of magnitude of the mean metabolite concentration in glucose-fed *E. coli*, we find that a linear model using NPSA and NCA predicts concentrations to within an order of magnitude with a significantly higher ∼80% accuracy (Materials and Methods and Figure S3). The difference between the measured concentrations and those predicted by our linear model is about 5-fold on average. This variation can be attributed to other global or local factors which affect metabolite concentrations. Also, error inherent to the measurement procedures limits the accuracy of the fit between model and data set.

Could the observed correlations stem from a systematic bias in the extraction and measurement procedures, which might prefer polar and charged metabolites over non-polar and un-charged ones? Indeed, some of the published data sets were obtained using extraction methods which risk losing lipophilic metabolites. For example, the two-phase water/chloroform extraction system used by Ishii *et al*. [9] may be biased towards the extraction of hydrophilic compounds. In order to control for such extraction biases and calibrate the intracellular metabolite concentrations, most studies spiked internal standards directly into the extraction fluid. Bennett *et al*. [8] and Fendt *et al*. [11] took the most stringent approach and added known concentrations of labeled standards of all compounds measured to the extraction solvent. Consequently, cellular metabolites and internal standards experienced the same opportunities for adsorptive losses or degradation [15]. This methodology enabled the authors to minimize sources of bias in the extraction and measurement procedures, indicating that the observed trends are unlikely to be the result of experimental artifacts (see Text S1 for further discussion).

When we restrict our analysis to amino acids, we find a significantly higher correlation between their hydrophobicity and measured concentrations (Figure S4). For amino acids, NPSA (or LogP) yields an R^2^ of more than 0.3 in all data sets, and in several cases it even surpasses 0.5. This trend is apparent when using LogP instead. We note that the concentration differences between free amino acids span two or three orders of magnitude. This large range cannot be explained by the well-known observation that hydrophobic amino acids are less abundant in proteins by about an order of magnitude [16].

The increased correlation observed for amino acids suggests that the observed trends might be more prominent when inspecting a group of metabolically similar compounds. Indeed, we find confirmation of this notion in phosphorylated nucleic acids, the concentrations of which correlate with NCA with R^2^>0.4, where each additional phosphate group increases concentration roughly three-fold on average. The observation that trends sharpen for groups of metabolically similar compounds suggests that the observed preference for polar, charged metabolites is present at multiple scales of inquiry and is indeed systematic.

There are, however, metabolites which display a consistent deviation from predicted concentrations. Most significant deviations from predicted concentrations occur only in specific conditions or data sets. Notably, glutamate and, to a lesser extent, glutamine are the only non-cofactor metabolites with MW<300 that display a consistent deviation from concentrations predicted using the four main physico-chemical parameters (NPSA, LogP, LogS and NCA) across most data sets. The concentration of glutamate is >30-fold higher than predicted, which has been explained by its role as a cellular nitrogen donor and counter-ion to potassium [8]. Notably, glutamate and glutamine can be regarded as co-factors, serving as nitrogen donors for the biosynthesis of essentially all other amino-acids.

## Discussion

Why should the concentration of hydrophobic, un-charged metabolites be lower in living cells? We hypothesize that concentrations are governed by evolutionary constraints. Here, we summarize and shortly discuss several previously suggested selective pressures acting in cells and how they might account for the observed trends.

A cellular preference for low hydrophobicity and high NCA can be attributed to a selection for decreased membrane permeability [17]. High permeability can result in metabolite leakage [17] or in metabolite accumulation within the membrane, which can lead to membrane instability [18]. Indeed, lipophilicity has become an important criterion in the pharmaceutical industry for estimating the permeability of small molecules through the intestinal membrane and their potential for use as oral drugs [17], [19]. In contrast, charged molecules are orders of magnitude less permeable as compared to their un-charged counterparts [20]. However, previous studies demonstrated that the negative effect of polar surface area (PSA) on permeability is considerably higher than the positive effect of NPSA [17]. As PSA does not exhibit consistent correlation with concentrations, permeability can only provide a partial explanation of the observed trends.

Another explanation for generally lower concentrations of hydrophobic metabolites is that non-polar and un-charged small compounds are at the risk of forming large colloid-like “aggregates” within the cell [21], [22]. These aggregates have been shown to enhance protein unfolding [23], and many synthetic aggregating compounds begin to aggregate at the low µM concentrations [22]. Furthermore, different compounds may promote aggregation synergistically when present in the same mixture [24]. Indeed, it has been shown that lipophilicity, solubility and lack of charged atoms are the most central factors determining the tendency of a compound to form aggregates [21].

Finally, a reduction in the concentration of non-polar metabolites can serve to decrease non-specific binding. Hydrophobic compounds can bind non-specifically to hydrophobic surfaces within the cells, including enzymatic active sites [25], [26], protein surfaces that participate in protein-protein interactions, or even nucleic acid strands [27]. Such hydrophobic stickiness is also associated with promiscuous activity of enzymes towards substrates other than their natural ones [28]. Indeed, in a study examining a large set of enzymes, the lipophilicity of a substrate was found to correlate with its participation in promiscuous drug binding [29]. According to this line of reasoning there is selective pressure to decrease the concentrations of metabolites that are highly hydrophobic and able to bind non-specifically to hydrophobic surfaces. Strengthening this explanation, a selection against non-specific binding of proteins and peptide ligands was demonstrated in the cellular protein interaction network of yeast [30].

We note that each of the above hypotheses actually refers to the phenomenon known as the hydrophobic effect: the preference of hydrophobic surfaces in an aqueous environment to adhere to other hydrophobic surfaces [31]. The “aggregation” hypothesis relates to self-adhesion while the “hydrophobic stickiness” and “membrane permeability” hypotheses refer to adhesion to other hydrophobic surfaces in the cell, the latter involving a specific hydrophobic organelle: the membrane. However, when hydrophobic metabolites are present in low enough concentrations, they are much less likely to diffuse out, aggregate, or bind non-specifically. That is, the “cost” of a metabolite, considering the above constraints, is a function of its concentration as well as its physico-chemical parameters.

From this perspective it is clear that the selective pressures we discuss do not necessarily predict a correlation between absolute concentrations and physico-chemical parameters relating to hydrophobicity. Rather, they predict a correlation when the absolute concentrations are high enough that the costs imposed by the various constraints discussed above are not negligible. In this light it is striking that we observe the significant level of correlation that we do, as several of the metabolites measured are present in extremely low concentrations (<10^−6^M), likely low enough to not be significantly affected by any of the above constraints. Conversely, a metabolite that is found in high concentration must be soluble and polar enough to meet the constraints imposed by the aqueous environment of the cell or it will certainly impose the costs we have described.

In conclusion, our study suggests that the concentrations of metabolites within the cell is not only a result of specific metabolic effects (i.e. kinetic parameters of the enzymes utilizing them), but also follows systematic global trends. Various large metabolomics data sets have accumulated in recent years and their number is predicted to increase rapidly as the technology improves and becomes more accessible. We believe that our study could raise the interest of the scientific community in the general questions addressed here and pave the way for future and more elaborate analysis. Such future studies could test and refine our findings and pinpoint the exact forces that shape the *in-vivo* concentrations of metabolites. Of special interest are the questions we addressed only partially: what is the relative importance of each of the discussed selective pressures? How do the differences between the internal environments of different organisms and organelles affect their distributions of metabolite concentrations? Do the constraints associated with different organisms and environments translate into preferences for different, parallel metabolic pathways, each employing different metabolites? We believe that the methodology put forward in this study enables inquiry into these questions and provides a better understanding of the forces shaping cellular life.

## Materials and Methods

### Obtaining the physico-chemical parameters

The physico-chemical parameters for all compounds analyzed are given in Dataset S1.

We used Pybel, the Python wrapper for OpenBabel (http://openbabel.sourceforge.net) to calculate the molecular mass, number of hydrogen bond acceptors, number of hydrogen bond donors, number of charged atoms and number of rotatable bonds [32]. Using the same software package we corrected all compounds to be in the protonation level most abundant at pH 7. The total hydrogen bond inventory of the molecule [33] was taken as hydrogen bond donors + hydrogen bond acceptors. The number of rotatable bonds refers to the internal molecule bonds that are able to freely rotate in solution but become restricted on passing from a free to a bound state, resulting in an entropic cost [34].

The molecular 3D-structure, essential for determining the surface area of the molecules, was also estimated using OpenBabel. We used *asa.py* (http://boscoh.com/protein/asapy) [35] to calculate the *total* surface area of the 3D-structure. We used the solvent-excluded surface area, representing the “cavity” the molecule creates in bulk solvent [36]. We also computed the *polar* surface area, i.e. the area contributed by polar atoms only (oxygen, nitrogen and the hydrogen atoms attached to them). The *non-polar* surface area is the difference between total surface area and polar surface area.

The logarithm of the octanol-water (LogP) partition coefficient for un-ionized compounds, was estimated using three different programs: XLOGP3 [37], ALogPS [38] and SciFinder (https://scifinder.cas.org/scifinder). In the paper, we use the ALogPS values since they were found to have the lowest RMSE for small molecules [37] and indeed they produce higher overall correlations. LogS, the logarithm of the solubility in water, was also estimated using ALogPS [38].

### Statistical analysis

We calculated the correlation between the metabolite concentrations in each data set and each of the physico-chemical parameters. For each such calculation, metabolites that were not measured in a given data set or did not have a value for that parameter, were discarded. To find a p-value for each R^2^ we used a Monte-Carlo permutation test. We created a distribution of randomized R^2^ values by shuffling the parameter values, randomly assigning them to metabolites and then correlating shuffled values with concentrations. We repeated this process 10^5^ times. The p-value was defined to be the fraction of times for which the randomized R^2^ values were higher than the original R^2^. To account for multiple hypothesis testing, we used false discovery rate (FDR) control [39], with a rate of 0.01 (n = 168, 21 data sets X 8 physico-chemical parameters).

### Predicting metabolite concentrations

Metabolite concentrations were predicted using least-squares multiple linear regression of log10 concentrations against the metabolite NPSA and NCA values. As before, high molecular weight compounds were removed from the analysis. In order to avoid potential over-fitting, the concentration of each metabolite was predicted using a model trained on all other metabolites and excluding the one to be predicted. As we are interested in global trends in concentration, the accuracy of the prediction was taken to be the fraction of predictions within an order of magnitude of the true concentration. In order to quantify the predictive power of our model, we compared the prediction accuracy to the accuracy of predicting the mean concentration for a given data set. For the case of glucose-fed *E. Coli* from Bennet *et. al*. we found that 78% of predictions were within one order of magnitude of the true concentrations while only 57% of measured concentrations were within one order of magnitude of the mean concentration (Figure S3).

## Supporting Information

Text S1 A computational analysis which suggests that a systematic bias in the extraction procedure is unlikely to account for the observed correlation between metabolite concentrations and NPSA (non-polar surface area) and NCA (number of charged atoms).
(DOC)

Dataset S1 Metabolite concentrations as measured in various organisms and conditions alongside the physicochemical parameters of all metabolites.
(XLS)

Figure S1 Correlation (R) between the logarithm of metabolites concentrations in each data set and the physico-chemical parameters of metabolites. All metabolites, (MW<300 & MW>300), were included in this analysis. A correlation that was found to be significant is denoted by *. See Figure 2.
(PDF)

Figure S2 Physico-chemical parameters significantly correlate with the logarithm of the metabolite concentrations in E. coli, as measured by Ishii et al. [9]. Median was taken across all repetitions. Metabolites are ordered (top to bottom) by increasing concentration. Physico-chemical parameters are ordered based on their correlation with concentrations, from the most negative correlation on the left to the most positive correlation on the right. Compound properties were normalized by subtracting the mean and dividing by the standard deviation, enabling consistent color coding of their values. R2 values are given at the top of the columns. p-values were calculated as described in the Methods, where ** correspond to a p-value<10-4 and * to a p-value<10-2. Parameter abbreviations are as in Figure 1.
(PDF)

Figure S3 Fraction of metabolites whose concentrations, as measured by Bennett et al. [8] (Glucose grown), is within a given factor of the prediction. Two predictions are used: the overall concentration mean and a linear regression using NPSA and NCA.
(PDF)

Figure S4 Correlation (R) between the logarithm of metabolites concentrations in each data set and the physico-chemical parameters of metabolites. Only amino-acids were included in this analysis. A correlation that was found to be significant is denoted by *. See Figure 2.
(PDF)
