# Supplementary material for: Hydrophobicity and Charge Shape Cellular Metabolite Concentrations
Source: PLoS Comput Biol. 2011 Oct 6;7(10):e1002166. doi: 10.1371/journal.pcbi.1002166 (PMC3188480; doi:10.1371/journal.pcbi.1002166)
Supplement: Text S1 — A computational analysis which suggests that a systematic bias in the extraction procedure is unlikely to account for the observed correlation between metabolite concentrations and NPSA (non-polar surface area) and NCA (number of charged atoms). (DOC) [file pcbi.1002166.s001.doc]

Hydrophobicity and Charge Shape Cellular Metabolite Concentrations

SUPPLEMENTARY INFORMATION

**Could the observed correlation between concentrations and NPSA and NCA stem from a systematic bias in the extraction procedure?**

The extraction procedure might prefer polar and charged metabolites over non-polar and un-charged ones, giving rise to the observed patterns. In the main text we suggest that this is unlikely to explain the observed trends as systematic differences were minimized by the use of labeled standards [1,2]. However, in spite of these controls, it is still possible that the adsorptive losses of metabolites and standards will differ considerably. For example, compounds within the cell might have greater opportunity to stick to the membrane as compared to external standards, which mostly experience the outer side of the membrane and only briefly encounter the inner side after the cell is lysed.

We analyzed the possible magnitude of this bias for the measured compounds with MW≤300. Assuming that the entire trend is the result of the adsorption of non-polar, un-charged metabolites on cell debris (mostly membrane) our null hypothesis is that the actual concentration of all metabolites is similar to that of the highly polar and charged ones. We therefore can estimate how much of each metabolite was adsorbed. To calculate the estimated adsorptive loss of a specific metabolite (in Bennett *et al.* data set [1]) we subtracted its measured concentration from the median concentration of the top 20% most polar and charged metabolites (~2.5 mM). Summing all estimated adsorptive losses of all the other 80% metabolites (46 metabolites) suggests that, overall, roughly 80 mM were lost. This is an estimate of the differential adsorptive losses (the difference between the adsorption of the metabolites and that of the standards) required to explain the trends. We think that such a massive differential loss of metabolites is improbable. Moreover, in each study there were many un-measured metabolites whose concentration would have to be lost in the same manner, thereby increasing the total required adsorption on the membrane.

To analyze the quantitative plausibility of an 80 mM adsorption we consider the relative mass that would need to be lost via adsorption on the membrane. Assuming the volume of *E. coli* to be ~1 µm^3^ = 10^-15^ L, the total metabolite loss of 80 mM corresponds to 8·10^-17^ mol, which is roughly 10^-14^ g (considering an average MW of 150 Da). The dry weight of *E. coli* is ~2·10^-13^ g [3]. The membrane, the main adsorptive component, comprises ~7% of *E. coli*’s dry weight [4], which is ~10^-14^ g. Therefore, in order to account for the observed trend, the membrane must adsorb a mass roughly equal to its own weight. Furthermore, as stated above, 10^-14^ g refers only to the measured compounds. We conclude that is it unlikely that the observed trends are the result of a difference between the adsorptive loss of metabolites and standards.

**REFERENCES**

1. Bennett BD, Kimball EH, Gao M, Osterhout R, Van Dien SJ, et al. (2009) Absolute metabolite concentrations and implied enzyme active site occupancy in Escherichia coli. Nat Chem Biol 5: 593-599.

2. Fendt SM, Buescher JM, Rudroff F, Picotti P, Zamboni N, et al. (2010) Tradeoff between enzyme and metabolite efficiency maintains metabolic homeostasis upon perturbations in enzyme capacity. Mol Syst Biol 6: 356.

3. Bremer H, Dennis P (1987) Modulation of chemical composition and other parameters of the cell by growth rate. Escherichia coli and Salmonella.

4. Nelson DL, Cox MM (2004) Lehninger Principles of Biochemistry: W. H. Freeman & Co.
