# Supplementary figures and images for: Hydrophobicity and Charge Shape Cellular Metabolite Concentrations
Source: PLoS Comput Biol. 2011 Oct 6;7(10):e1002166. doi: 10.1371/journal.pcbi.1002166 (PMC3188480; doi:10.1371/journal.pcbi.1002166)

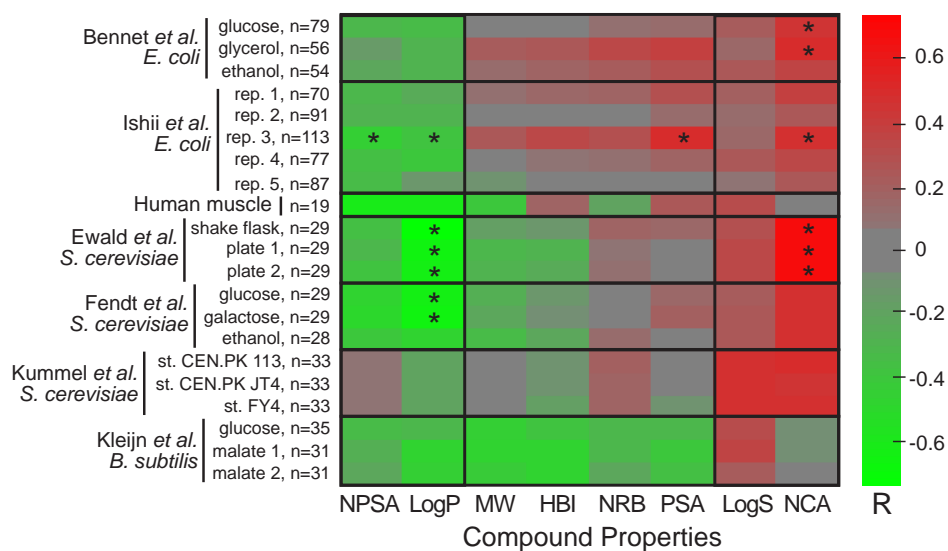

Supplement: Figure S1 — Correlation (R) between the logarithm of metabolites concentrations in each data set and the physico-chemical parameters of metabolites. All metabolites, (MW<300 & MW>300), were included in this analysis. A correlation that was found to be significant is denoted by *. See Figure 2. (PDF) [file pcbi.1002166.s003.pdf]

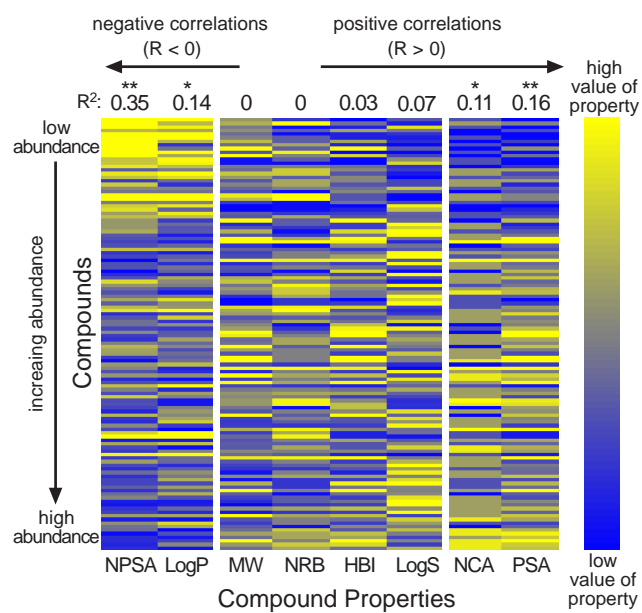

Supplement: Figure S2 — Physico-chemical parameters significantly correlate with the logarithm of the metabolite concentrations in E. coli, as measured by Ishii et al. [9]. Median was taken across all repetitions. Metabolites are ordered (top to bottom) by increasing concentration. Physico-chemical parameters are ordered based on their correlation with concentrations, from the most negative correlation on the left to the most positive correlation on the right. Compound properties were normalized by subtracting the mean and dividing by the standard deviation, enabling consistent color coding of their values. R2 values are given at the top of the columns. p-values were calculated as described in the Methods, where ** correspond to a p-value<10-4 and * to a p-value<10-2. Parameter abbreviations are as in Figure 1. (PDF) [file pcbi.1002166.s004.pdf]

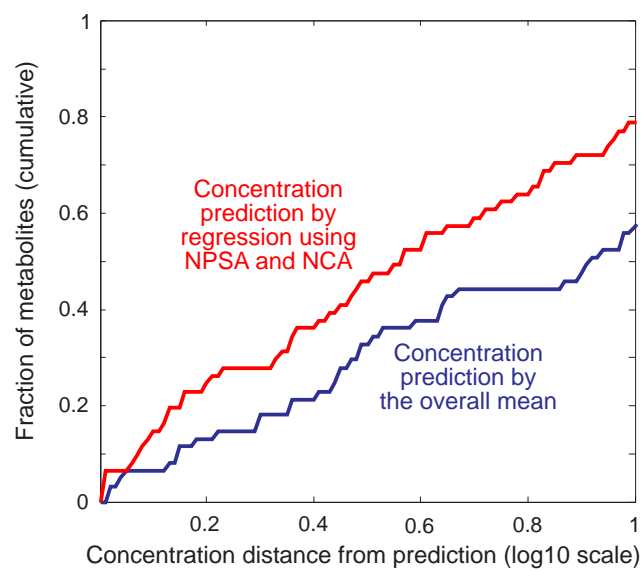

Supplement: Figure S3 — Fraction of metabolites whose concentrations, as measured by Bennett et al. [8] (Glucose grown), is within a given factor of the prediction. Two predictions are used: the overall concentration mean and a linear regression using NPSA and NCA. (PDF) [file pcbi.1002166.s005.pdf]

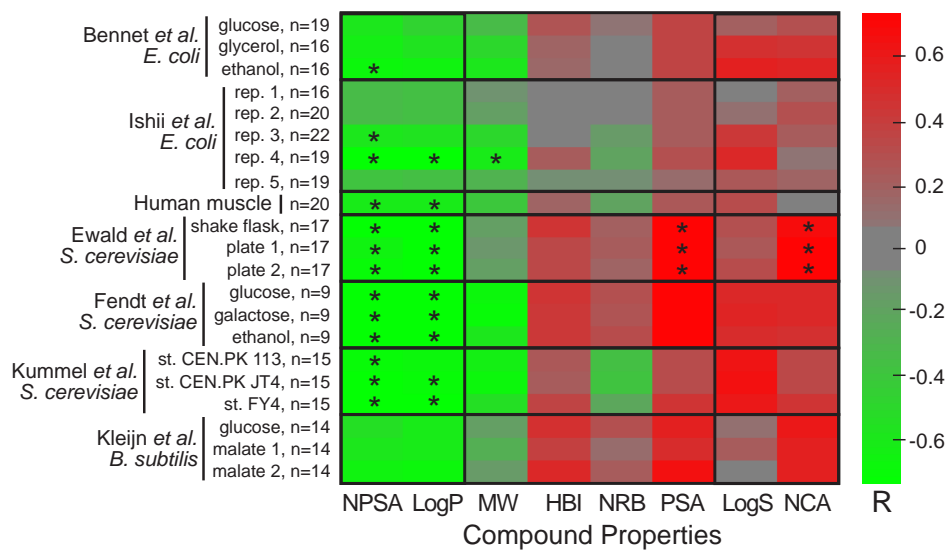

Supplement: Figure S4 — Correlation (R) between the logarithm of metabolites concentrations in each data set and the physico-chemical parameters of metabolites. Only amino-acids were included in this analysis. A correlation that was found to be significant is denoted by *. See Figure 2. (PDF) [file pcbi.1002166.s006.pdf]
